# U. S. V. M. A.

**Published:** 1898-02

**Authors:** 


### U. S. V. M. A.

Chairman Butler, of the Executive Committee, reports the decision of that body in favor of Omaha for the place of meeting in September, 1898.

President Salmon announces the following additional appointments for the year's work. The Army Legislative Committee has been increased to five instead of three, as in former years. He has continued Dr. J. P. Turner at the head of the Army Legislative Committee:

*Committee on Army Legislation:* Dr. J. P. Turner, Fort Meyer, Va., Chairman; Dr. M. Stalker, Ames, Ia.; Dr. A. G. Vogt, Newark, N. J.; Dr. Austin Peters, Boston, Mass.; Dr. E. P. Niles, Blacksburg, Va.

*Resident Slate Secretaries.*

| | |
|---|---|
| Alabama | C. A. Cary, Auburn. |
| Arizona | J. C. Norton, Phœnix. |
| Arkansas | R. R. Dinwiddie, Fayetteville. |
| California | Fred. C. Pierce, 1724 Webster St., Oakland. |
| Colorado | D. P. Frame, 11 South Wasatch Ave., Colorado Springs. |
| Connecticut | R. P. Lyman, 328 Asylum St., Hartford. |
| Delaware | H. P. Eves, 507 W. Ninth St., Wilmington. |
| District of Columbia | A. M. Farrington, Department of Agriculture, Washington. |
| Georgia | George P. Blackman, Rome. |
| Illinois | F. S. Schoenleber, Morris. |
| Indiana | J. R. Mitchell, Evansville. |
| Iowa | T. A. Brown, Chariton. |
| Kansas | R. H. Harrison, Atchison. |
| Kentucky | F. S. Eisenman, 222 East Main St., Louisville. |
| Louisiana | W. H. Dalrymple, Baton Rouge. |
| Maine | William S. Lord, U. S. Hotel, Portland. |
| Maryland | Wm. Dougherty, 1035 Cathedral St., Baltimore. |
| Massachusetts | J. F. Winchester, Lawrence. |
| Michigan | S. Brenton, 83 Fifth St., Detroit. |
| Minnesota | M. H. Reynolds, St. Anthony Park. |
| Mississippi | J. C. Roberts, Agricultural College. |
| Missouri | Charles Ellis, 3230 Locust St., St. Louis. |
| Montana | M. E. Knowles, Butte. |
| Nebraska | W. D. Hammond, Wayne. |
| New Hampshire | Lemuel Pope, Jr., Portsmouth. |
| New Jersey | J. P. Lowe, Bloomfield Ave., Passaic. |
| New York | William Henry Kelly, 195 Western Ave., Albany. |
| North Carolina | A. S. Wheeler, Biltmore. |
| North Dakota | T. D. Hinebauch, Fargo. |
| Ohio | T. Bent Cotton, Mount Vernon. |
| Pennsylvania | W. H. Ridge, Trevose. |
| Rhode Island | Walter L. Burt, 26 Tabor Avenue, Providence. |
| South Carolina | Benjamin McInnes, Charleston. |
| South Dakota | M. J. Treacy, Fort Meade. |
| Tennessee | Joseph Plaskett, 529 Broad St., Nashville. |
| Texas | M. Francis, College Station. |

| | |
|---|---|
| Virginia . . . . . | E. P. Niles, Blacksburg. |
| Washington . . . . | S. B. Nelson, Pullman. |
| West Virginia . . . | L. N. Reefer, 1406 Chapline St., Wheeling. |

